# Development of the Olfactory Epithelium and Nasal Glands in TMEM16A^-/-^ and TMEM16A^+/+^ Mice

**DOI:** 10.1371/journal.pone.0129171

**Published:** 2015-06-11

**Authors:** Devendra Kumar Maurya, Tiago Henriques, Monica Marini, Nicoletta Pedemonte, Luis J. V. Galietta, Jason R. Rock, Brian D. Harfe, Anna Menini

**Affiliations:** 1 Laboratory of Olfactory Transduction, SISSA, International School for Advanced Studies, Trieste, Italy; 2 Istituto Giannina Gaslini, Genova, Italy; 3 Department of Anatomy, UCSF School of Medicine, San Francisco, CA, United States of America; 4 Department of Molecular Genetics and Microbiology Genetics Institute, University of Florida, College of Medicine, Gainesville, FL, United States of America; Monell Chemical Senses Center, UNITED STATES

## Abstract

TMEM16A/ANO1 is a calcium-activated chloride channel expressed in several types of epithelia and involved in various physiological processes, including proliferation and development. During mouse embryonic development, the expression of TMEM16A in the olfactory epithelium is dynamic. TMEM16A is expressed at the apical surface of the entire olfactory epithelium at embryonic day E12.5 while from E16.5 its expression is restricted to a region near the transition zone with the respiratory epithelium. To investigate whether TMEM16A plays a role in the development of the mouse olfactory epithelium, we obtained the first immunohistochemistry study comparing the morphological properties of the olfactory epithelium and nasal glands in TMEM16A^-/-^ and TMEM16A^+/+^ littermate mice. A comparison between the expression of the olfactory marker protein and adenylyl cyclase III shows that genetic ablation of TMEM16A did not seem to affect the maturation of olfactory sensory neurons and their ciliary layer. As TMEM16A is expressed at the apical part of supporting cells and in their microvilli, we used ezrin and cytokeratin 8 as markers of microvilli and cell body of supporting cells, respectively, and found that morphology and development of supporting cells were similar in TMEM16A^-/-^ and TMEM16A^+/+^ littermate mice. The average number of supporting cells, olfactory sensory neurons, horizontal and globose basal cells were not significantly different in the two types of mice. Moreover, we also observed that the morphology of Bowman’s glands, nasal septal glands and lateral nasal glands did not change in the absence of TMEM16A. Our results indicate that the development of mouse olfactory epithelium and nasal glands does not seem to be affected by the genetic ablation of TMEM16A.

## Introduction

TMEM16A/ANO1, a member of the family of transmembrane proteins with unknown function 16 [1,2], has been recently identified as a calcium-activated chloride channel [3–5]. TMEM16A is expressed in several types of cells of secretory epithelia, smooth muscle cells [6–8], as well as in cells of sensory systems: cochlea [9–10], retina [11–13], nociceptive neurons [14–15], vomeronasal sensory epithelium [11,16–17], and olfactory epithelium [11,16,18]. TMEM16A is involved in several types of physiological processes [6–7] including proliferation and development.

A role of TMEM16A in proliferation had been already suggested before its identification as a calcium-activated chloride channel. Indeed, TMEM16A was reported to be overexpressed in some malignant tumors and was known by different names, such as DOG1 (Discovered On Gastrointestinal stromal tumor protein 1 [19–20]), TAOS2 (Tumor Amplified and Overexpressed Sequence 2 [21]) overexpressed in oral squamous cell carcinomas, and ORAOV2 (Oral Cancer Overexpressed 2 [22]) overexpressed in oral and esophageal squamous cell carcinomas. In addition to a potential role for TMEM16A in proliferation, suggested by the overexpression of this channel in some tumors, TMEM16A has also been shown to be a regulator of cell proliferation in healthy cells. Indeed, Stanich et al [23] showed that TMEM16A regulates proliferation of interstitial cells of Cajal at the G_1_/S transition of the cell cycle.

Some studies also indicated a possible role of TMEM16A in the development of the trachea [24] and the cochlea [10]. Rock et al [24] showed that TMEM16A is expressed in the epithelium of the developing trachea and in the embryonic tracheal muscle of mice. Furthermore, the same authors produced knockout mice for TMEM16A and showed that these mice have alterations in the formation of tracheal cartilage rings and die within one month, possibly because of tracheomalacia. In addition to providing a mouse model of tracheomalacia, these results point out to the possible role of TMEM16A in epithelial and smooth muscle cell organization in development [24]. Reduced transepithelial current and accumulation of mucus in the trachea of these mice indicate that TMEM16A also play a role in secretory processes [25,26]. Additional alterations caused by TMEM16A loss of function include block of gastrointestinal peristalsis and reduced nociception [15,27].

Another study [10], suggested that TMEM16A plays a developmental role in the mouse postnatal developing cochlea. Indeed, these authors showed that supporting cells in the greater epithelial ridge of the cochlea exhibited spontaneous calcium-dependent volume changes that were inhibited by anion channel blockers, indicating that volume changes may be related to the activity of calcium-activated chloride channels. Moreover, volume changes were correlated with the time course and location of TMEM16A expression in the cochlea, suggesting that TMEM16A may be the pacemaker of spontaneous activities in postnatal developing cochlea.

Based on previous studies showing that TMEM16A plays a role in cell proliferation and in development [7,10,24] and on our previous observation that at embryonic day E12.5 TMEM16A immunoreactivity was present at the apical surface of the entire olfactory epithelium, whereas from E16.5 TMEM16A immunoreactivity was restricted to a region near the transition zone with the respiratory epithelium [18], we investigated whether TMEM16A plays a role in the development of the olfactory epithelium. For this purpose, we used immunohistochemistry to identify morphological properties of the olfactory epithelium and nasal glands during mouse embryonic development and at postnatal age in TMEM16A^+/+^ and TMEM16A^-/-^ mice.

## Materials and Methods

### Ethics Statement

All animals were handled in accordance with the Italian Guidelines for the Use of Laboratory Animals (Decreto Legislativo 27/01/1992, no. 116) and European Union guidelines on animal research (No. 86/609/EEC). Experimental procedures were notified to and approved by the Italian Ministry of Health, Directorate General for Animal Health. The work has been performed on the explanted tissues from sacrificed mice and did not require ethical approval, as stated by the Italian law (decree 116/92). The entire procedure is in accordance with the regulations of the Italian Animal Welfare Act, with the relevant EU legislation and guidelines on the ethical use of animals and is approved by the local Authority Veterinary Service.

Experiments were performed on TMEM16A^-/-^ and TMEM16A^+/+^ littermate mice obtained by breeding TMEM16A^+/-^ mice generated by Rock et al. [24]. Male and female mice were put together for mating in the evening and separated the next morning. If a vaginal plug was observed in the morning, that day was designated as embryonic day 0.5 (E0.5). Once the mouse was positive for vaginal plug, on the prerequisite embryonic day the mouse was anaesthetized by CO_2_ inhalation, followed by cervical dislocation. Embryos were removed from the uterus and decapitated. The head region was further processed for immunohistochemistry. For postnatal mice, date of birth was defined as postnatal day 0 (P0). Postnatal mice were anaesthetized by CO_2_ inhalation and decapitated. Nose was separated from the rest of head and further processed.

### Genotyping protocol

To check the genotype of mouse for *Tmem16a* gene, genotyping for deletion of exon-12 of *Tmem16a* and insertion of PGK-neo cassette was done. Genomic DNA was isolated from the mouse tails by using 5'PRIME Kit (Eppendorf, Milano, Italy), according to manufacturer’s protocol. PCR was carried out in a total volume of 25 μl under the following conditions for 40 cycles: 94°C for 5 min (for 1 cycle), 94°C for 30 sec, 60°C for 30 sec and 72°C for 30 sec. The final reaction mixture contained 100 ng of genomic DNA, using Taq Polymerase Master Mix (VWR, Milano, Italy). Two separate PCRs were required to identify homozygous knockout mouse: one for the mutant allele (PGK-neo instead of exon-12) and one for the wild type allele. Wild type allele size was 330 bp and mutant allele was 450 bp. DNA was separated by electrophoresis on 1.5% agarose gel with ethidium bromide.

Primers used:

WT (f): 5’-CCTATGACTGCCAGGGACGCCC-3’
WT(rev): 5’-TGTTCCTGTCCCTGCAATGCGG-3’
Mut (f): 5’-GACGCCCTCCATTGACCC-3’
Mut (rev): 5’-GCAGTAGAAGGTGGCGCGAAG-3’

### Immunohistochemistry

For E12.5 and E14.5 whole head region and for E16.5, E18.5 and P4 dissected out nose was fixed in 4% paraformaldehyde prepared in 0.01 M phosphate-buffered saline (PBS) for overnight at 4°C. Tissues with olfactory epithelium were equilibrated at 4°C in 30% (wt/vol) sucrose until the tissue sank to base in solution for cryoprotection. Then the tissue was embedded in O.C.T. (Bio-optica, Milano, Italy) and stored at −80°C. Before sectioning on cryostat, O.C.T. blocks were kept at −20°C for at least 12 hours. With a cryostat, 12–14 μm coronal sections were cut and stored (-80°C) for further use. For antigen retrieval, sections were usually treated with SDS 0.5% (wt/vol) in PBS for 15 min. However, for the following primary antibodies: cytokeratin 8, sox2, Ki67, and cytokeratin 5, heat-induced antigen retrieval was used. Sections in 0.01 M citrate buffer (pH 6.0) were heated in a microwave oven for 20 min. After cooling, sections were rinsed three times in PBS. Sections were incubated in a blocking solution [2% FBS (vol/vol) and 0.2% (vol/vol) Triton X-100 in PBS] for 90 min, and then with the primary antibody (diluted in the blocking solution) overnight at 4°C. Sections were then rinsed with 0.1% (vol/vol) Tween 20 in PBS (PBS-T) and incubated with the fluorophore-conjugated secondary antibody (diluted in PBS-T) for 2 h at room temperature. After washing with PBS-T, sections were treated with 0.1 μg/ml DAPI for 30 min, washed with PBS-T, and mounted with Vectashield (Vector Laboratories, Burlingame, CA). Postnatal mice tissues were fixed for 6 hours and processed as described for the embryonic tissues. As far as possible, different embryonic and postnatal tissues were processed in parallel at the same time to avoid any discrepancies in results.

For each age, sections were analyzed from at least three mice obtained from at least two litters.

All chemicals, unless otherwise stated, were purchased from Sigma, Milano, Italy.

The primary antibodies used in this study are listed in Table 1.

**Table 1:** Primary antibodies used in this study.

Primary antibody	Immunogen	Dilution	Manufacturer/catalog number/lot number or clone
Rabbit polyclonal TMEM16A	Synthetic peptides corresponding to amino acid residues 424–519, 628–731 and 904–986 of human TMEM16A	1:50	Abcam/ab53212/GR71118-3
Goat polyclonal TMEM16A	Synthetic peptide corresponding to amino acid residues 825–875 of human TMEM16A	1:50	Santa Cruz Biotech/sc-69343/ H0713
Rabbit polyclonal adenylyl cyclase III (ACIII)	Synthetic peptide corresponding to amino acid residues 1125–1144 of human ACIII	1:100	Santa Cruz Biotech/sc-588/ K0608
Goat polyclonal olfactory marker protein (OMP)	Purified natural rat OMP	1:1000	Wako Chemicals/ 544-10001/ IUP1001
Mouse monoclonal ezrin	Synthetic peptide corresponding to amino acid residues 362–585 of human ezrin	1:100	Abcam/ab4069/3C12
Rabbit monoclonal cytokeratin 8	Synthetic peptide corresponding to amino acid residues 300–350 of human cytokeratin 8	1:150	Novus Biologicals/NB110-56919/EP1628Y
Rabbit polyclonal aquaporin 5	Synthetic peptide corresponding to 17 amino acid sequence in the cytoplasmic region of rat aquaporin 5	1:150	Calbiochem/178615/D00140208
Goat polyclonal sox2	Synthetic peptide corresponding to amino acid residues 277–293 of human sox2	1:50	Santa Cruz Biotech/sc-17320/ A1314
Goat polyclonal Ki67	Synthetic peptide mapping near the C-terminus of Ki67 of mouse origin	1:150	Santa Cruz Biotech/ sc-7846/ C2012
Rabbit polyclonal cytokeratin 5	Synthetic peptide sequence derived from the C-terminus of the mouse keratin 5 protein	1:200	BioLegend/ 905501/ D14FF0122

### Secondary antibodies

The following secondary antibodies obtained from Invitrogen (Eugene, OR, USA), were used: donkey anti-rabbit Alexa Fluor 488 (1:500; catalog no. A21206), donkey anti-goat Alexa Fluor 594 (1:500; catalog no. A11058), goat anti-rabbit Alexa Fluor 594 (1:500; catalog no. A11037), goat anti-mouse Alexa Fluor 488 (1:500; catalog no. A11001)

### Imaging

Immunoreactivity was usually visualized with a confocal microscope (TCS SP2; Leica) using 40X/1.25NA or 63X/1.4NA oil immersion objectives. Images were acquired using Leica software (at 1,024 × 1,024 pixel resolution). All images were taken as average of z-stacks of 1–2 μm thickness. Images in Fig 7 were obtained with a Nikon Eclipse 90i microscope using a Plan Apo VC 60X/1.4NA oil immersion objective. Images were prepared and assembled in Inkscape version 0.48.2. Images were not modified other than to level illumination. Only DAPI signals were enhanced, to show the anatomy of the olfactory epithelium. In any case, data were not altered because of the above adjustments.

### Cell counting

To determine cell density, the number of nuclei in a 150 x 150 μm^2^ area of the olfactory epithelium was counted with imageJ 1.48v software. As nuclei of the olfactory epithelium have various shapes, we counted the cells manually using the cell counter tool of imageJ 1.48v. Eight coronal sections of 16 μm thickness were collected from each animal. For E14.5 every fourth section was collected, while for E16.5, E18.5 and P4 every seventh section was collected. Approximately 40–47 areas were selected from the septum and turbinates in each animal to count the cells. Areas were chosen randomly at different epithelial thicknesses. In each group at least three animals were used.

Number of cells are reported as average ± SEM. Statistical significance was determined using paired or unpaired Student’s *t*-tests and p values <0.05 were considered significant.

## Results

**Fig 1:**
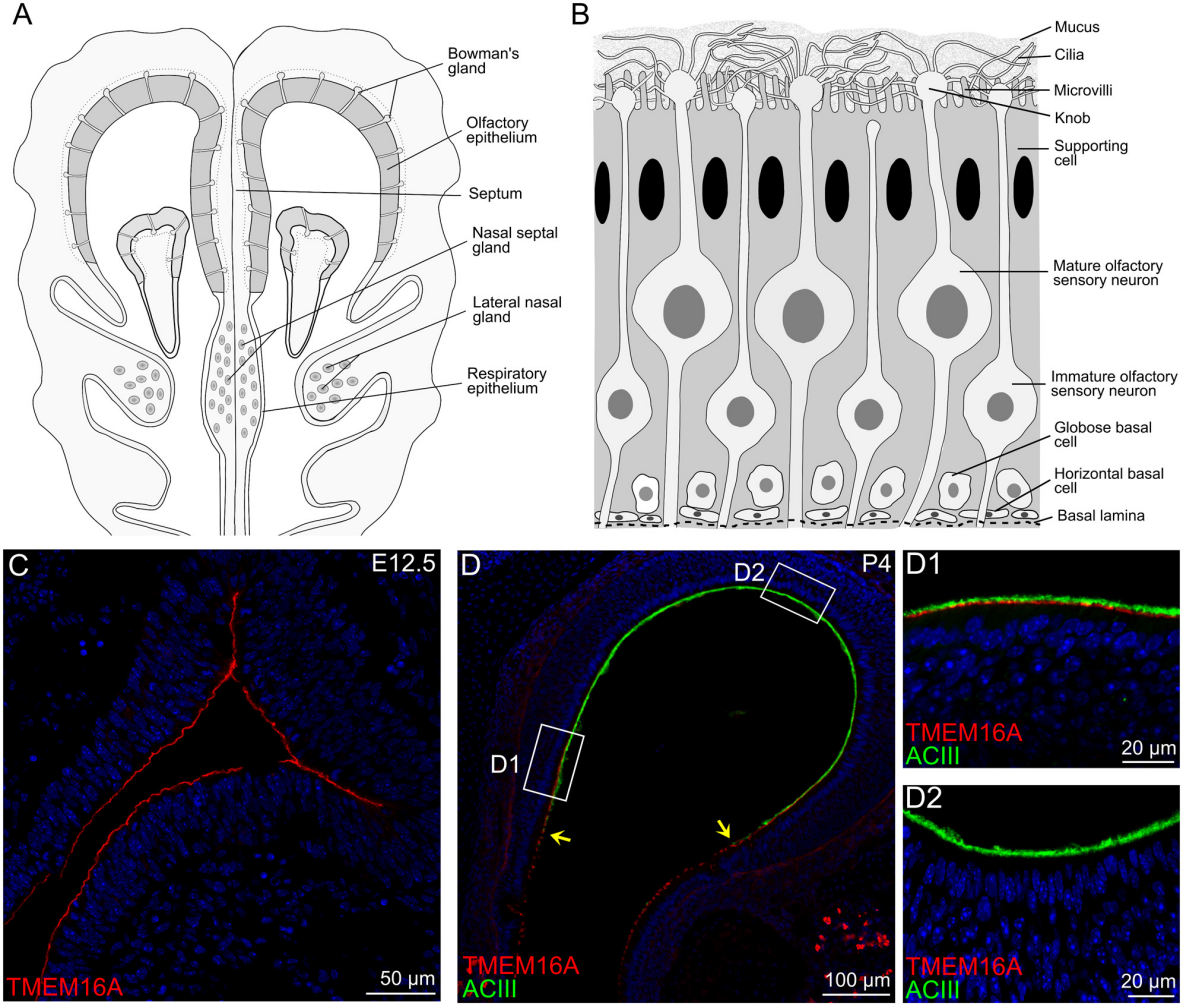
Schematic representations and confocal images of nose coronal sections. **A:** Schematic diagram of a nose coronal section showing the olfactory epithelium, respiratory epithelium, Bowman’s glands, nasal septal glands, and lateral nasal glands. **B:** The pseudostratified olfactory epithelium is mainly composed of supporting cells, olfactory sensory neurons and basal cells. Supporting cells have columnar cell bodies, microvilli at the apical side, and reside in the most apical region of the epithelium. Mature and immature olfactory sensory neurons are bipolar neurons with a single dendritic process projecting toward the apical surface of the epithelium. In mature olfactory sensory neurons, several cilia protrude from the dendritic knob. Two types of basal cells reside near the basal lamina: horizontal basal cells (HBCs) that are attached to the basal lamina, and globose basal cells (GBCs) that are located above the HBC layer. **C-D:** confocal images of coronal sections of the olfactory epithelium at E12.5 and P4. **C:** At E12.5, TMEM16A was expressed at the surface of the entire olfactory epithelium. **D:** At P4, TMEM16A immunoreactivity was present only in the olfactory regions toward the respiratory epithelium. Arrows in (**D)** indicate the transition between olfactory and respiratory epithelium. The apical surface of the olfactory epithelium was well stained by adenylyl cyclase III (ACIII). Higher magnification images taken from the boxed areas are shown in D1 and D2. Cell nuclei were stained by DAPI. Scale bars: C = 50 μm; D = 100 μm; D1-D2 = 20 μm.

Fig 1A shows a schematic diagram of a nose coronal section illustrating the localization of the olfactory epithelium, respiratory epithelium, and various types of glands. The olfactory epithelium is mainly composed of olfactory sensory neurons, supporting cells, and basal cells, as schematized in Fig 1B.

In agreement with our previous observations [18], TMEM16A is expressed at E12.5 at the entire apical surface of the olfactory epithelium (Fig 1C), whereas at P4 it is expressed only in a region of the olfactory epithelium near the transition zone with the respiratory epithelium [Fig 1(D, D1, D2)]. Moreover, Gritli-Linde et al [28] showed a dynamic expression of *Tmem16a* during embryonic development, with the highest expression at E12.5, which greatly decreased after E18.5. TMEM16A has been shown to be involved in various physiological processes, including cell proliferation and development. In the developing olfactory epithelium, mitotic cells are abundant at the apical surface at E12.5 [29,30], and the majority of mitoses occur in the apical layer up to about E14, whereas later proliferative activity is transferred to the basal layer [31,32]. Based on these observations, we investigated the hypothesis that TMEM16A may play a role in cell proliferation and development of the mouse olfactory epithelium by comparing results obtained with TMEM16A^-/-^ and TMEM16A^+/+^ littermates.

### Expression of ACIII and OMP in TMEM16A^-/-^ and TMEM16A^+/+^ mice

**Fig 2:**
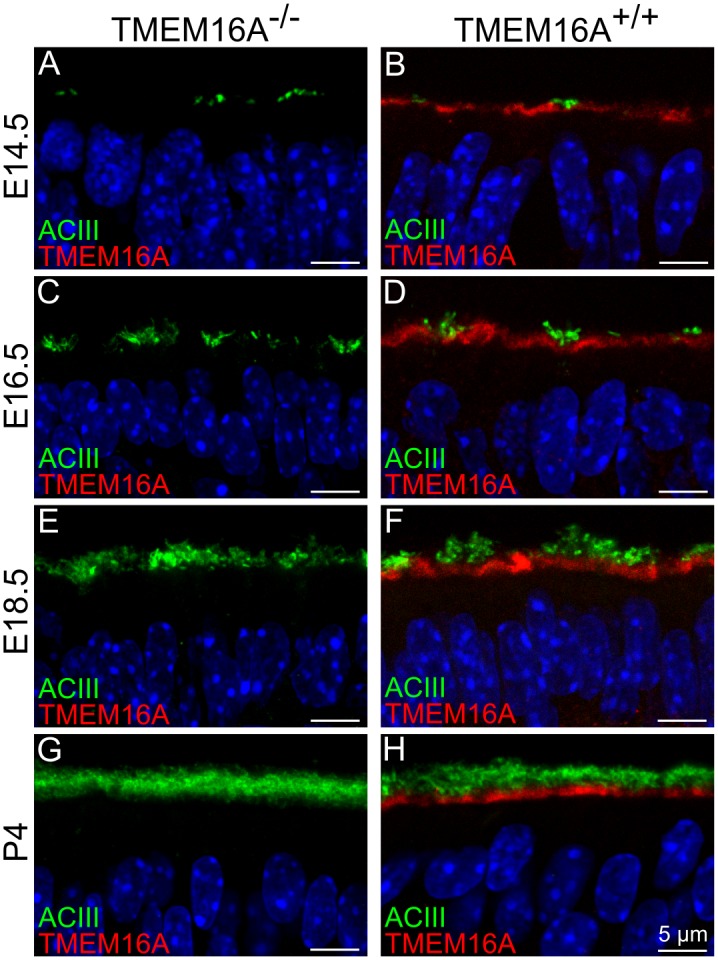
Expression of TMEM16A and ACIII in the olfactory epithelium of TMEM16A^-/-^ and TMEM16A^+/+^ littermate mice. Confocal images of coronal sections of the olfactory epithelium from a region near the transition zone with the respiratory epithelium at E14.5, E16.5, E18.5 and P4. **A, C, E, G:** No immunoreactivity to TMEM16A was detectable in TMEM16A^-/-^ mice (goat anti-TMEM16A). **B, D, F, H:** TMEM16A expression in TMEM16A^+/+^ mice was below adenylyl cyclase III (ACIII) expression and did not overlap with ACIII. TMEM16A immunostaining is discontinuous because of interruption by dendritic knobs of olfactory sensory neurons. Expression of ACIII was similar in both types of mice. Images are averages of z-stacks of ~1.0 μm thickness. Cell nuclei were stained by DAPI. Scale bars = 5 μm.

In a first set of experiments, we investigated the expression of TMEM16A and ACIII, a well-known ciliary marker protein in olfactory sensory neurons [33–35]. In the olfactory epithelium of TMEM16A^-/-^ mice, TMEM16A immunoreactivity was absent, thus confirming the loss of TMEM16A and the specificity of the antibody for this protein, while ACIII was expressed at the apical surface of the olfactory epithelium (Fig 2).

In TMEM16A^+/+^ mice, Fig 1C and 1D and Fig 2 confirm our previous results [18], showing that TMEM16A was expressed at E12.5 at the apical surface of the entire olfactory epithelium (Fig 1C), whereas from E16.5 to postnatal age it was expressed only in a region of the olfactory epithelium near the transition zone with the respiratory epithelium (Fig 1D and Fig 2). Fig 2B shows that at E14.5 both ACIII and TMEM16A were expressed at the apical surface of the olfactory epithelium, with ACIII present in small clusters and TMEM16A expressed in a layer just below the ACIII clusters (Fig 2B). At subsequent days of development ACIII immunoreactivity increased and at P4 the two antibodies stained distinct layers at the apical surface without any overlap (Fig 2H).

The expression of ACIII in TMEM16A^-/-^ and TMEM16A^+/+^ littermates between E14.5 and P4 appears to be largely similar (Fig 2A–2H). As ACIII is expressed in the cilia of olfactory sensory neurons, genetic ablation of TMEM16A does not seem to cause any large change in the development of the ciliary layer in the olfactory epithelium.

**Fig 3:**
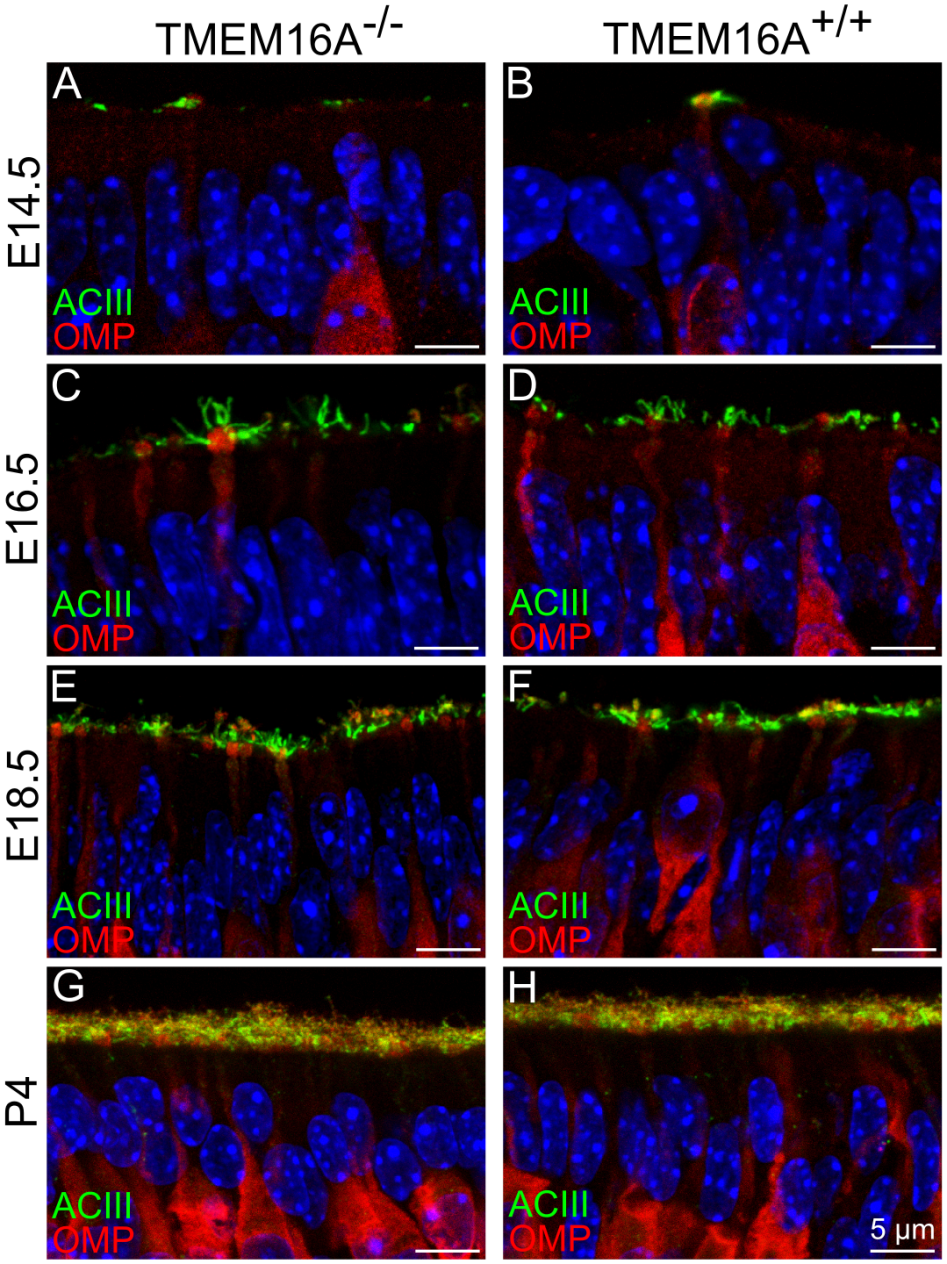
Olfactory sensory neurons in the developing olfactory epithelium of TMEM16A^-/-^ and TMEM16A^+/+^ mice. Mature olfactory sensory neurons express the olfactory marker protein (OMP). Confocal images of coronal sections of the olfactory epithelium at E14.5, E16.5, E18.5 and P4 from TMEM16A^-/-^ (**A, C, E, G**) or TMEM16A^+/+^ (**B, D, F, H)** mice. In both mice, at E14.5 a limited number of mature neurons was present (**A, B**), but the number progressively increased from E16.5 to P4 (**C-H**). ACIII signals were seen in the cilia protruding from the dendritic knob of mature olfactory sensory neurons (**A-H**). Mature neurons expressing OMP and cilia marked by ACIII were similar in TMEM16A^-/-^ and TMEM16A^+/+^ littermates. Images are averages of z-stacks of ~1.5 μm thickness. Cell nuclei were stained by DAPI. Scale bars = 5 μm.

To further evaluate whether TMEM16A has an influence on the maturation of olfactory sensory neurons during development, we used the olfactory marker protein (OMP) as the typical marker for mature olfactory sensory neurons [36]. A small number of OMP immunopositive neurons were present at E14.5 both in TMEM16A^-/-^ and TMEM16A^+/+^ embryos (Fig 3A and 3B). With development, the density of mature neurons increased and firmly packed OMP positive neurons were present at postnatal stage in both types of mice (Fig 3A–3H).

These results indicate that genetic ablation of TMEM16A does not seem to affect the maturation of neurons and cilia in the olfactory epithelium (Figs 2 and 3).

### Supporting cells in TMEM16A^-/-^ and TMEM16A^+/+^ mice

**Fig 4:**
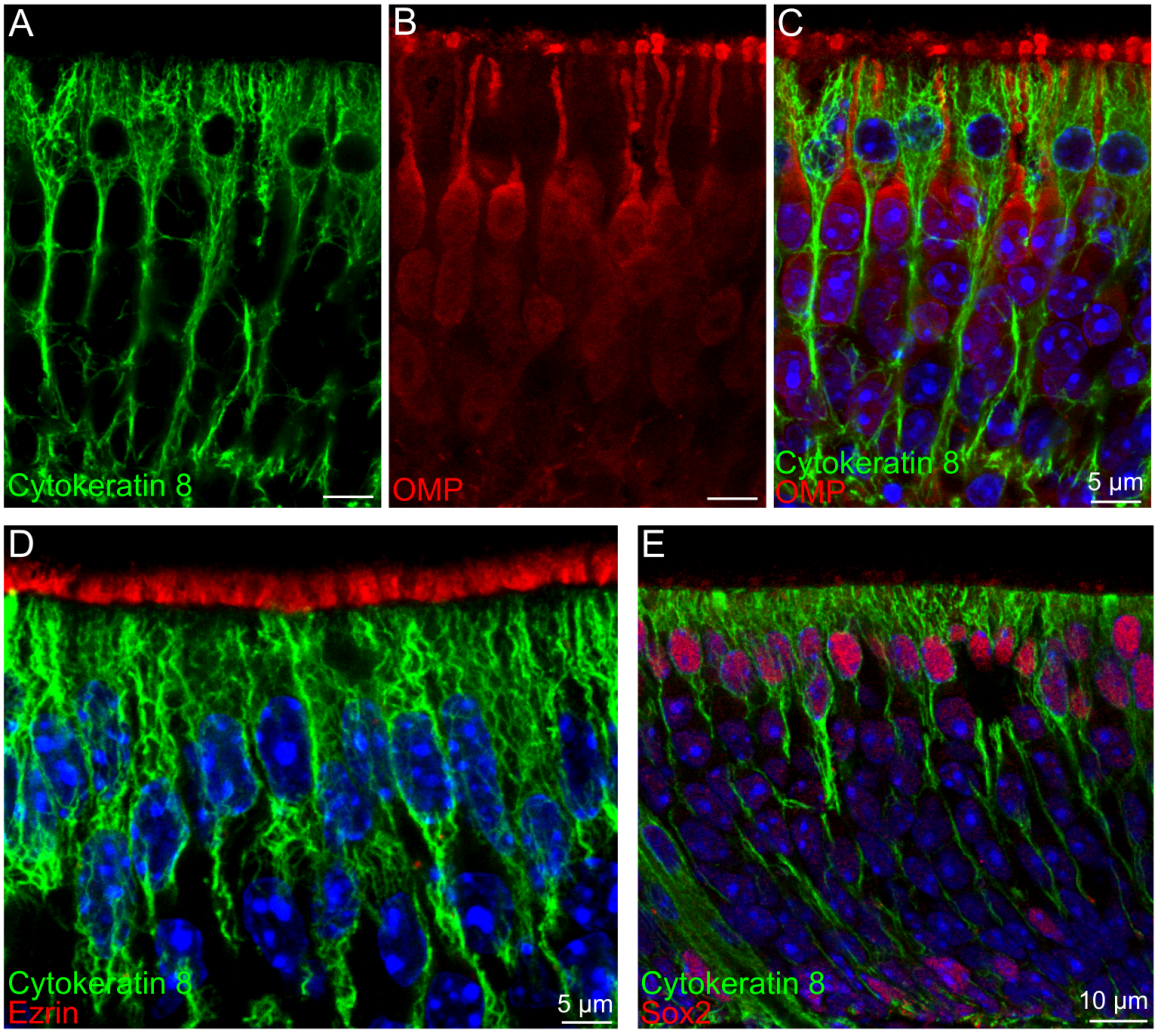
Markers for supporting cells. **A-C:** Cytokeratin 8 marked cells with the typical morphology of supporting cells. Double staining of cytokeratin 8 with OMP showed no co-localization. **D:** Microvilli of supporting cells stained by ezrin were not stained by cytokeratin 8. **E:** Double staining of cytokeratin 8 with sox2 shows that sox2 is a nuclear marker for supporting cells whose nuclei are located in the apical region of the epithelium. Sox2 also stains nuclei of basal cells. Coronal sections of the olfactory epithelium of wild type mice at P60 (**A-C**), or P4 (**D, E**). Images are averages of z-stacks of ~1.5 μm thickness. Cell nuclei were stained by DAPI. Scale bars: A-C, D = 5 μm; E = 10 μm.

We have previously reported that TMEM16A is localized at the apical part of supporting cells and in their microvilli [18]. Here, we investigated whether TMEM16A has an influence on the development of supporting cells. We used cytokeratin 8, ezrin and sox2 as markers for supporting cells. Fig 4A shows that cytokeratin 8, a cytoskeleton protein, stained cells with the typical morphology of supporting cells, characterized by large cell bodies with the shape of an inverted flask located in the apical region of the epithelium and processes reaching the basal part of the epithelium, although microvilli at the apical surface were not marked by cytokeratin 8. Furthermore, we performed a double-labeling experiment using OMP (Fig 4B) and found that cytokeratin 8 and OMP immunoreactivity did not overlap (Fig 4A–4C), indicating that cytokeratin 8 does not label olfactory neurons and is a good marker for supporting cells. Fig 4D further confirms that cytokeratin 8 immunoreactivity was absent in microvilli, as illustrated by the absence of overlap with ezrin immunoreactivity. Fig 4E shows that sox2 is a good marker for the nuclei of supporting cells, although it also stains nuclei of basal cells located near the basal lamina of the olfactory epithelium, in agreement with previous reports [37–39].

**Fig 5:**
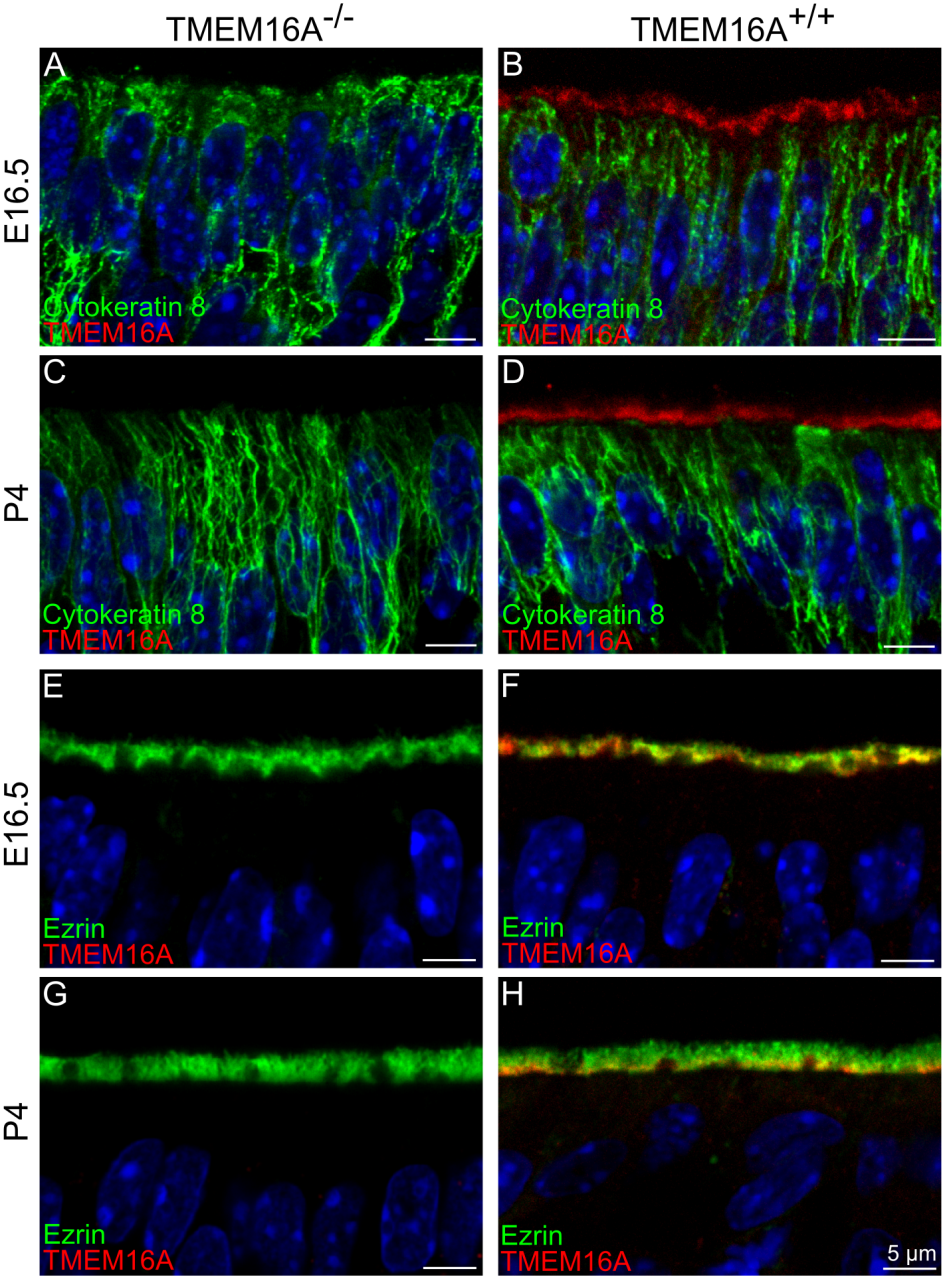
Expression of TMEM16A, cytokeratin 8 and ezrin in the olfactory epithelium of TMEM16A^-/-^ and TMEM16A^+/+^ littermate mice. Confocal images of coronal sections of the olfactory epithelium from a region near the transition zone with the respiratory epithelium at E16.5 and P4 from TMEM16A^-/-^ (**A, C, E, G**) or TMEM16A^+/+^ (**B, D, F, H)**. No immunoreactivity to TMEM16A was detectable in TMEM16A^-/-^ mice. **A-D:** Supporting cells marked by cytokeratin 8 were similar in both types of mice. In TMEM16A^+/+^ mice, TMEM16A (goat anti-TMEM16A) and cytokeratin 8 immunoreactivity did not overlap (**B, D**), whereas TMEM16A expression (rabbit anti-TMEM16A) partially overlapped with ezrin immunopositive signals (**F, H**). Supporting cells marked by cytokeratin 8 and microvilli marked by ezrin were similar in both types of mice. Images are averages of z-stacks of ~1.5 μm thickness. Cell nuclei were stained by DAPI. Scale bars = 5 μm.

To examine the anatomical organization of supporting cells in TMEM16A^-/-^ and TMEM16A^+/+^ littermates, we first stained the olfactory epithelium with cytokeratin 8. Fig 5A–5D shows large similarities in the organization of supporting cells. Furthermore, a comparison among microvilli of supporting cells marked by ezrin (Fig 5E–5H) also shows a similarity in TMEM16A^-/-^ and TMEM16A^+/+^ littermates. Moreover, Fig 5F and 5H confirms our previous observation that TMEM16A was mainly localized to the proximal part of microvilli of supporting cells and to the apical part of supporting cells [18].

### Quantitative comparison among cell types in TMEM16A^-/-^ and TMEM16A^+/+^ mice

To obtain a quantitative comparison among various cell types in the olfactory epithelium we counted supporting cells, neuronal cells, and basal cells in TMEM16A^-/-^ and TMEM16A^+/+^ mice. Moreover, since from E16.5 to postnatal age TMEM16A expression in the olfactory epithelium of TMEM16A^+/+^ mice is restricted to a region near the transition zone with the respiratory epithelium, we evaluated whether cell numbers change in different regions by comparing cell count analysis obtained in regions with and without TMEM16A expression. Corresponding regions were selected for cell counting in TMEM16A^-/-^ mice.

It is well known that sox2 stains both nuclei of supporting and basal cells and that these cells can be distinguished on the basis of their shape and position in the olfactory epithelium [37–39], as schematically shown in Fig 1B. To count supporting cells we considered oval-shaped nuclei stained by sox2 at the apical part of the olfactory epithelium (Fig 4E).

**Fig 6:**
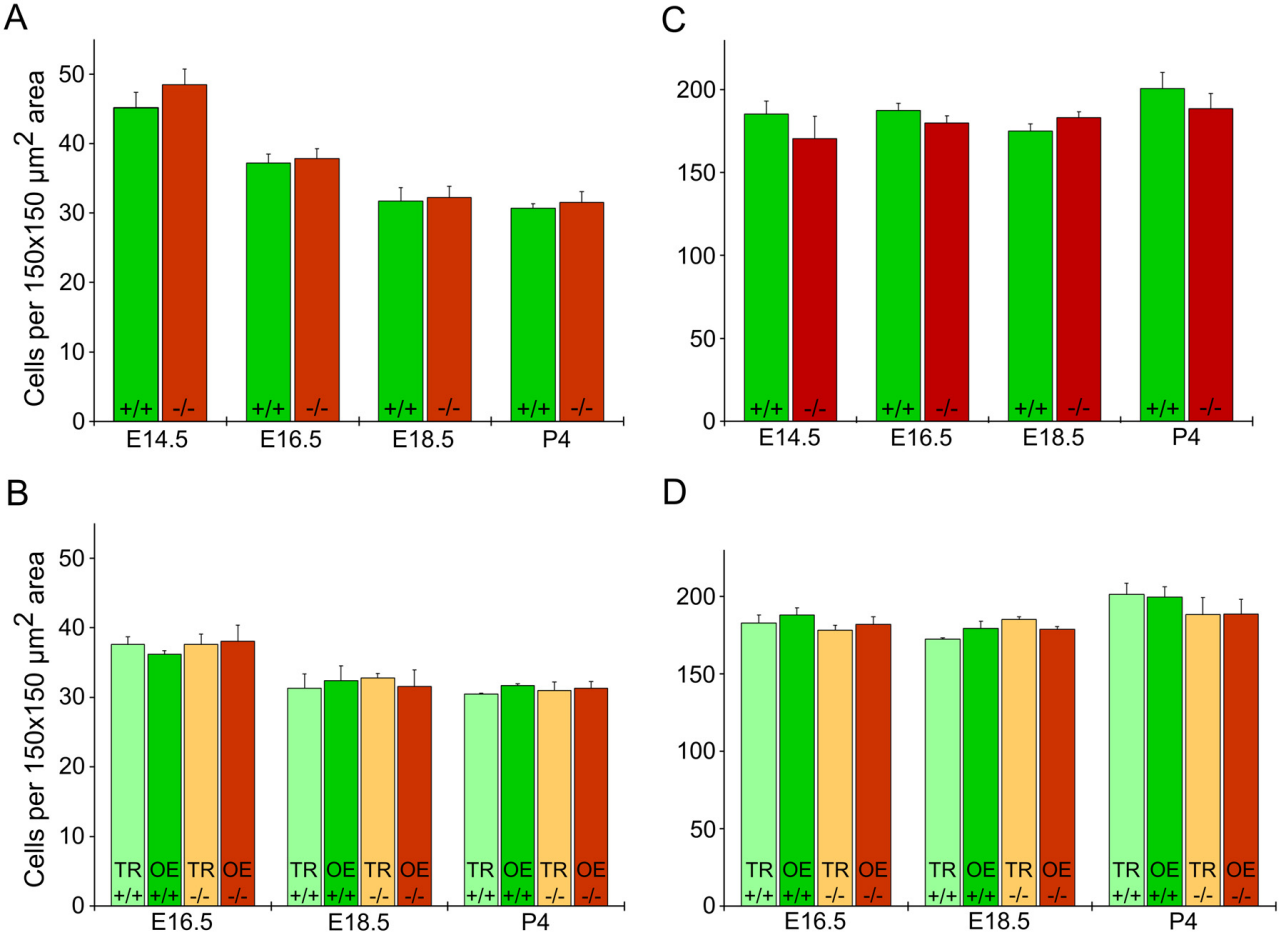
Cell densities in the olfactory epithelium of TMEM16A^-/-^ and TMEM16A^+/+^ littermate mice. Comparison among the average number of supporting cells and olfactory sensory neurons in the olfactory epithelium of TMEM16A^-/-^ and TMEM16A^+/+^ littermate mice. Average number of supporting cells (**A, B**) or neuronal cells (**C, D**) was calculated by counting nuclei in 150 x 150 μm^2^ areas from several regions of the olfactory epithelium. **B, D:** Average number of cells calculated near the transition zone with the respiratory epithelium (TR), corresponding to TMEM16A expression in TMEM16A^+/+^ mice, or far from the transition zone (OE). Counting was done in three different animals for each group and presented as average ± SEM.

The average number of supporting cells at E14.5 was 45 ± 2 in TMEM16A^+/+^ mice, not significantly different from the value of 48 ± 2 in TMEM16A^-/-^ mice (p-value > 0.05, Fig 6A). It may be of interest to note that the average number of supporting cells decreased as the age increased, reaching the value of 32 ± 2 at E18.5 for both TMEM16A^+/+^ and TMEM16A^-/-^ mice (p-value < 0.05, Fig 6A). At every age, the average number of supporting cells was not significantly different between TMEM16A^+/+^ and TMEM16A^-/-^ mice (Fig 6A). For ages between E16.5 and P4, we also compared the average number of supporting cells in regions of the olfactory epithelium near and far from the transition zone with the respiratory epithelium and did not find a significant difference, as shown in Fig 6B.

To obtain a quantitative estimate of olfactory sensory neurons (both mature and immature), we counted nuclei that were not stained by sox2 and were located in the middle layer, between the basal and the apical region of the olfactory epithelium. The average number of olfactory sensory neurons at E14.5 was 185 ± 8 in TMEM16A^+/+^ mice, not significantly different from the value of 170 ± 14 in TMEM16A^-/-^ mice (Fig 6C). At every age, the average number of neurons was not significantly different between TMEM16A^+/+^ and TMEM16A^-/-^ mice (Fig 6C). As observed for supporting cells for ages between E16.5 and P4, also the average number of olfactory sensory neurons in regions of the olfactory epithelium near and far from the transition zone with the respiratory epithelium was not significant different, as shown in Fig 6D.

**Fig 7:**
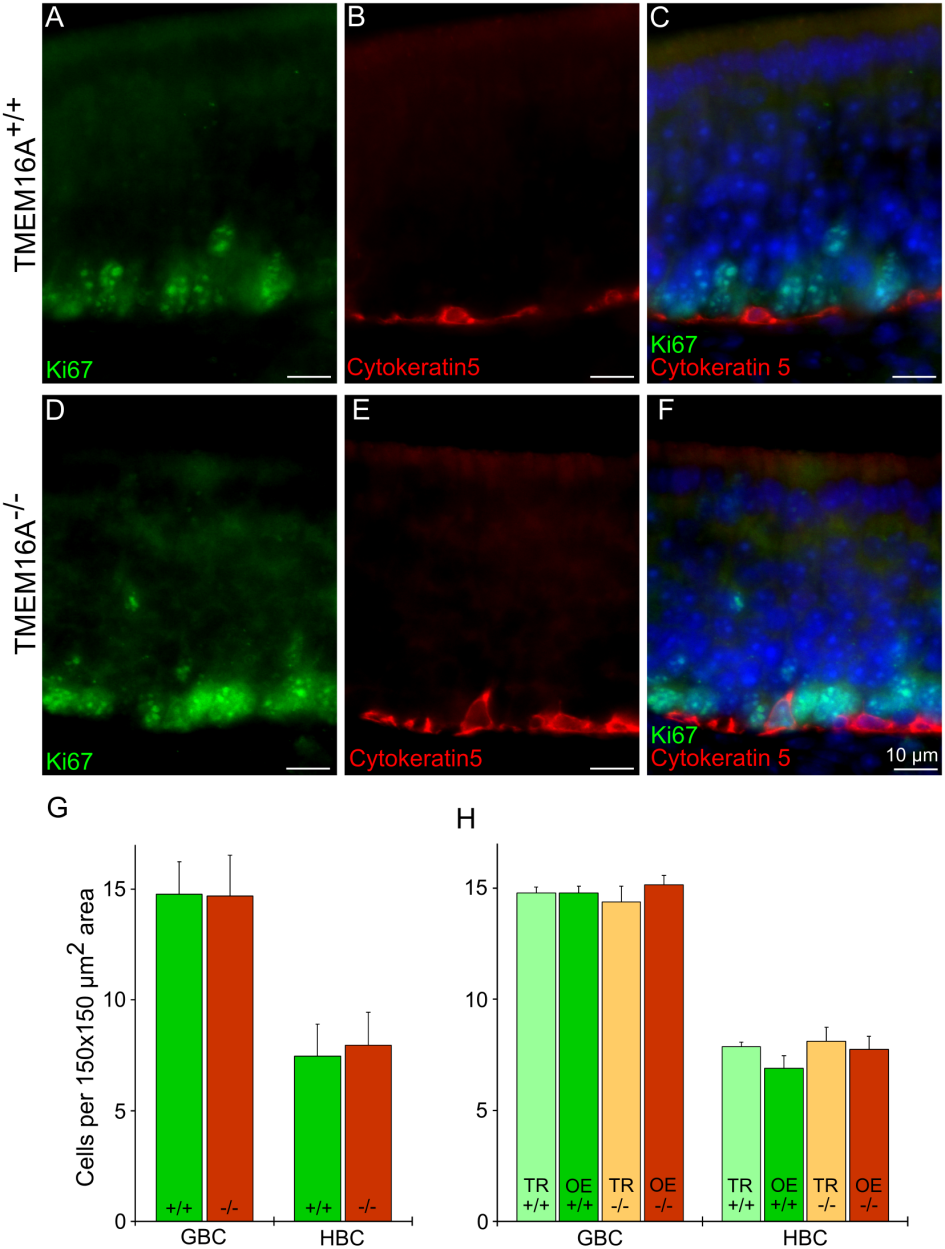
Expression of cytokeratin 5 and Ki67 in the olfactory epithelium of TMEM16A^-/-^ and TMEM16A^+/+^ littermate mice. Confocal images of coronal sections of the olfactory epithelium from a region near the transition zone with the respiratory epithelium at P4 from TMEM16A^+/+^ (**A-C**) and TMEM16A^-/-^ (**D-F)** mice. Cell nuclei were stained by DAPI. Scale bars = 10 μm. **G:** Comparison among the average number of horizontal (HBC) and globose (GBC) basal cells in the olfactory epithelium of TMEM16A^+/+^ and TMEM16A^-/-^ littermate mice. Average numbers of HBCs or GBCs were calculated by counting nuclei in 150 x 150 μm^2^ areas from several regions of the olfactory epithelium. **H:** Average number of cells calculated near the transition zone with the respiratory epithelium (TR), corresponding to TMEM16A expression in TMEM16A^+/+^ mice, or far from the transition zone (OE). Counting was done in three different animals for each group and presented as average ± SEM.

Furthermore, we estimated the number of horizontal and globose basal cells at P4. Horizontal basal cells lie near the basal lamina, while globose basal cells are located above the layer of horizontal basal cells (Fig 1B). We used cytokeratin 5 to identify horizontal basal cells [40–41], and Ki67 to stain globose basal cells (Fig 7A–7F). Ki67 is a marker of proliferating cells in all phases of the proliferating cycle, except for G0, that is often used as a marker for globose basal cells [42–45].

At P4, the average number of horizontal basal cells was 7 ± 1 in TMEM16A^+/+^ mice, not significantly different from the value of 8 ± 1 in TMEM16A^-/-^ mice (Fig 7G). The average number of globose basal cells was 15 ± 1 in TMEM16A^+/+^ mice, not significantly different from the value of 15 ± 2 in TMEM16A^-/-^ mice (Fig 7G). Furthermore, we also compared the average numbers in regions near and far from the transition zone with the respiratory epithelium and did not find a significant difference, as shown in Fig 7H.

In addition, in agreement with previous studies, we found that very few horizontal basal cells are immunopositive for proliferating markers in the normal olfactory epithelium [40]. Indeed, out of 2787 cells stained by cytokeratin 5 in TMEM16A^+/+^ and TMEM16A^-/-^ mice we found only 16 cells (0.006%) co-stained by Ki67.

Taken together, these results indicate that genetic ablation of TMEM16A does not significantly affect the average number of olfactory sensory neurons and supporting cells during mouse embryonic development and at postnatal age. Moreover, the average number of horizontal basal cells and globose basal cells at P4 also remained similar in the presence and in the absence of TMEM16A.

### Bowman and nasal glands in TMEM16A^-/-^ and TMEM16A^+/+^ mice

We investigated the expression of TMEM16A in Bowman’s glands, nasal septal glands, and lateral nasal glands, whose localization is schematically represented in Fig 1A, and used aquaporin 5 as a marker for these glands [46].

**Fig 8:**
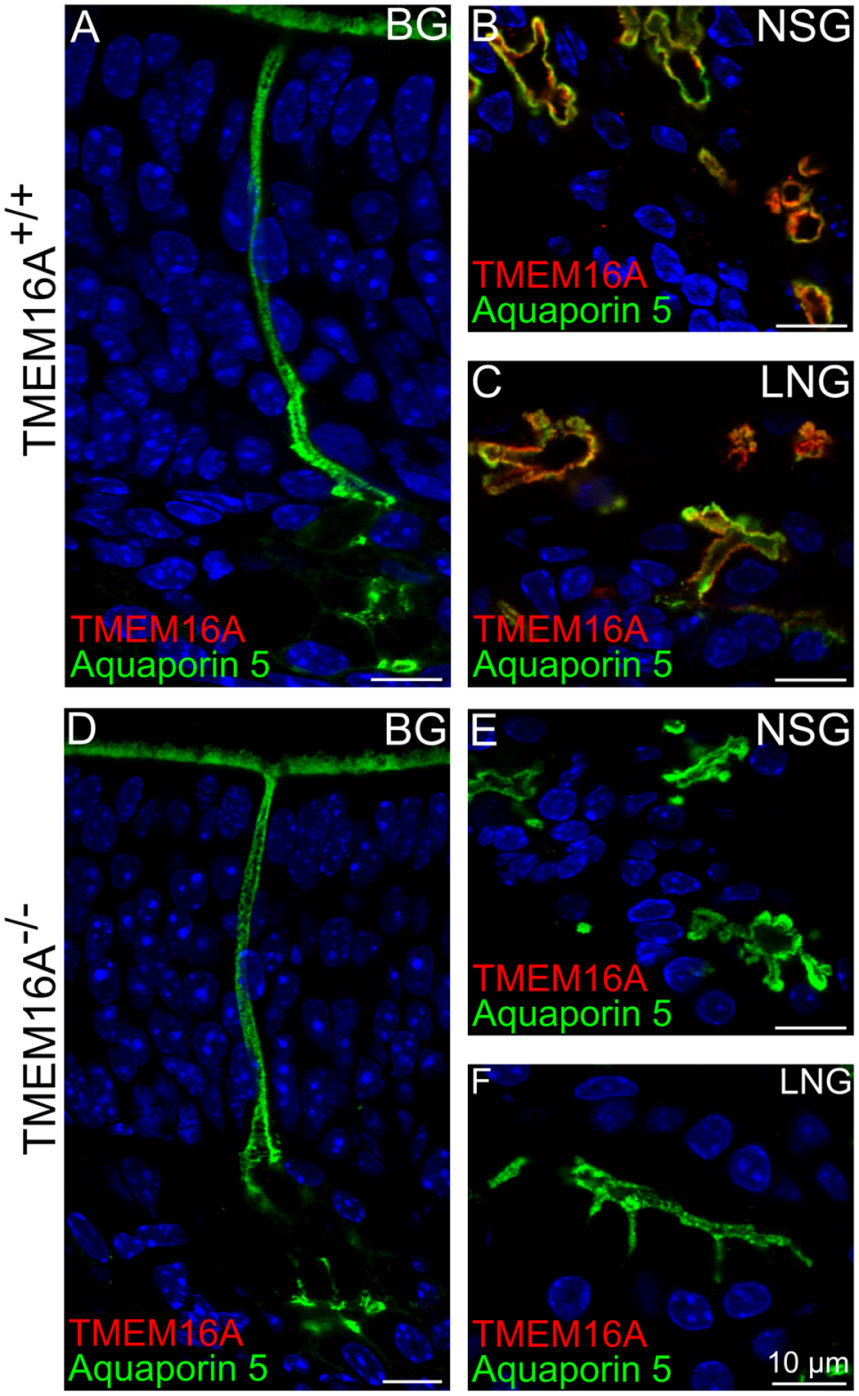
Expression of TMEM16A in various nasal glands of TMEM16A^-/-^ and TMEM16A^+/+^ littermate mice. Expression of TMEM16A and aquaporin 5 in the Bowman’s gland (BG), nasal septal gland (NSG) and lateral nasal gland (LNG) of TMEM16A^-/-^ and TMEM16A^+/+^ littermate mice. **A, D:** aquaporin 5 immunopositive signals were seen in Bowman’s glands in P4 mice. Aquaporin 5 expression in glands and ducts is clearly visible. TMEM16A immunoreactivity (goat anti-TMEM16A) was not present in Bowman’s glands of TMEM16A^-/-^ nor of TMEM16A^+/+^ littermate mice (**A, D)**. However, aquaporin 5 and TMEM16A were co-expressed in nasal septal glands (**B**) and lateral nasal glands (**C**) of TMEM16A^+/+^ mice. No immunoreactivity to TMEM16A was detectable in TMEM16A^-/-^ mice (**E, F**). Glands marked by aquaporin 5 were similar in both types of mice. Images are averages of z-stacks of thickness of ~2.0 μm for **A, D**, or ~1 μm for **B, C, E, F**. Cell nuclei were stained by DAPI. Scale bars = 10 μm.

Fig 8A and 8D shows that aquaporin 5 stained the internal wall of the duct of a Bowman’s gland as well as microvilli of supporting cells. Previous reports showed expression of TMEM16A in the duct of Bowman’s glands and in the luminal surface of nasal septal glands and lateral nasal glands [16]. However, in TMEM16A^+/+^ mice, we did not find expression of TMEM16A in Bowman’s glands, whereas the luminal surface of nasal septal glands and lateral nasal glands expressed both TMEM16A and aquaporin 5 (Fig 8B and 8C). A comparison with results from TMEM16A^-/-^ mice shows that immunostaining of aquaporin 5 remains unchanged, whereas TMEM16A immunoreactivity was absent (Fig 8D–8F). The morphology of Bowman’s gland, nasal septal glands and lateral nasal glands remained the same in both types of mice.

Thus, the absence of TMEM16A does not seem to influence the development of Bowman’s glands, nasal septal glands and lateral nasal glands.

## Discussion

In this study, we have obtained for the first time immunohistochemistry data comparing morphological and anatomical properties of the olfactory epithelium and of Bowman’s and nasal glands during mouse embryonic development and at postnatal ages in TMEM16A^+/+^ and TMEM16A^-/-^ mice.

### Olfactory sensory neurons and supporting cells

As TMEM16A^-/-^ mice die before reaching P30 and 90% of them die before P9, we restricted our study up to the age of P4. In the first part of this study, we showed that the development and the morphology of olfactory sensory neurons were similar between TMEM16A^+/+^ and TMEM16A^-/-^ mice. Interestingly, an *in vitro* study suggested that TMEM16A is involved in the early phase of ciliogenesis [47]. In the olfactory epithelium, we did not find expression of TMEM16A in the cilia of olfactory sensory neurons [18]. Furthermore, the similar morphology of cilia and knobs in TMEM16A^+/+^ and TMEM16A^-/-^ mice indicates that TMEM16A does not mediate ciliogenesis in olfactory sensory neurons.

To investigate whether TMEM16A has an influence on the development of supporting cells we first used cytokeratin 8 as a marker for supporting cells. Cytokeratin 8 or keratin 8 is a subtype of keratin intermediate filaments. It is type II (basic) keratin and is found associated with type I (acidic) cytokeratin 18 and cytokeratin 19 in various epithelial cells [48–50]. We found that cytokeratin 8 is a selective marker for supporting cells of the olfactory epithelium, indeed cytokeratin 8 immunostaining revealed cells with the typical morphology of supporting cells and, in addition, did not overlap with the immunopositive signals of OMP that stains mature olfactory sensory neurons. As cytokeratin 8 stains the cytoskeleton, its use allows the investigation of changes in supporting cell cytoskeleton during development. Both cytokeratin 8 and cytokeratin 18 are the abundant intermediate filament subtypes during mouse embryonic development [51]. Using cytokeratin 8 as a marker for supporting cell cytoskeleton, we found that at E14.5 the cytoskeleton was loosely organized and individual filaments of the cytoskeleton could be observed (data not shown). In postnatal mice, cytokeratin 8 immunopositive cytoskeletons became densely packed and supporting cells’ nuclei aligned in the top layer of the olfactory epithelium. In TMEM16A^+/+^ mice, TMEM16A expression was observed just above the cytokeratin 8 immunopositive signals, but we did not found any overlap in their expression (Fig 5). TMEM16A^-/-^ mice were devoid of TMEM16A expression, but cytokeratin 8 expression pattern remained very similar to that observed in TMEM16A^+/+^ littermate mice, indicating that organization and morphology of supporting cells were not largely affected by the absence of TMEM16A.

TMEM16A has been shown to interact with ezrin in salivary gland epithelial cells [52]. We confirmed our previous observation that TMEM16A was expressed in the apical part of supporting cells and in their apical microvilli marked by ezrin [18]. Double staining of TMEM16A with ezrin showed that at E16.5 TMEM16A and ezrin immunoreactivity largely overlapped, whereas at later ages TMEM16A was only expressed in the proximal part of microvilli. A very similar pattern of expression was observed in TMEM16A^-/-^ mice. A comparison between ezrin expression in microvilli in TMEM16A^+/+^ and TMEM16A^-/-^ littermate mice indicates that microvilli development did not seem to be largely altered by the absence of TMEM16A.

It is of interest to note that TMEM16A, ezrin and cytokeratin 8 expressed in supporting cells, but cytokeratin 8 immunoreactivity did not even partially overlap with TMEM16A or ezrin immunoreactivity (Fig 4D, and Fig 5A–5D). An explanation for the absence of overlap between cytokeratin 8 and TMEM16A may arise from the ultrastructural study of the morphology of supporting cells by Frisch [53]. Indeed, Frisch [53] reported that, at the apical part of supporting cells, the cytoplasm seems to be strongly gelated and devoid of cellular organelles. Indeed, we observed a space between ezrin and cytokeratin 8 immunoreactivity (Fig 4D), which resembles the apical organelle free area described by Frisch [53]. TMEM16A was expressed in the free apical region of supporting cells and in the proximal part of microvilli (Fig 5). A similar free space without cellular organelles just below microvilli has also been reported in intestinal brush border cells [54].

TMEM16A plays a role in cell proliferation in several systems. For example, proliferation of interstitial cells of Cajal in TMEM16A^-/-^ mice has been shown to be severely affected by the absence of TMEM16A [23]. In addition, TMEM16A is widely known for its role in carcinogenic tumor proliferation [19–22,55,56]. Several studies from cancerous cell lines showed that cell proliferation was severely affected by reducing TMEM16A expression with siRNA or inhibiting its activity by using TMEM16A blockers [23,57,58], although other studies showed that TMEM16A overexpression did not affect proliferation [55,58–60].

In the olfactory epithelium, Gritli-Linde et al [28] showed that *Tmem16a* expression was high at E12.5, but greatly decreased after E18.5. We observed TMEM16A expression in supporting cells all over the olfactory epithelium during early stages of development (E12.5 and E14.5), whereas from E16.5 onward TMEM16A expression became restricted toward the transition zone between the olfactory and the respiratory epithelium [18]. In the early stages of development dividing cells are abundant in the apical part of the olfactory epithelium [31,32] and TMEM16A may play a role in cell division. Based on these observations, we counted the number of supporting cells and of olfactory sensory neurons and found that at E14.5 the average number of supporting cells was significantly higher than average values at later stages of development. However, similar values were estimated in TMEM16A^+/+^ and TMEM16A^-/-^ littermate mice, indicating that TMEM16A did not play a role in this process.

The average number of olfactory sensory neurons was not significantly different from E14.5 to P4 and between TMEM16A^+/+^ and TMEM16A^-/-^ mice. In addition, the average number of globose basal cells and of horizontal basal cells at P4 remained similar in the two types of mice indicating that TMEM16A expression does not affect proliferation in the olfactory epithelium.

### Bowman’s and nasal glands

TMEM16A is expressed in various secretory epithelia and glands and regulates anion secretion [25–27,61–62]. The nasal cavity contains several types of secretory glands, such as Bowman’s glands, nasal septal glands and lateral nasal glands. The olfactory epithelium is directly exposed to changes in environment and prone to getting in contact with hazardous chemicals and stimuli. The apical part of the olfactory epithelium is always covered with mucus, which continuously eliminates the unwanted molecules from the surface. Mucus is primarily secreted by Bowman’s gland and supporting cells are involved in mucus secretion and ionic composition maintenance [53,63].

Aquaporin 5, a water channel, is expressed in Bowman’s gland and supporting cells’ microvilli [46,64]. We observed aquaporin 5 expression in the duct cells and secretory acinar cells of Bowman’s gland both in TMEM16A^+/+^ and TMEM16A^-/-^ littermate mice (Fig 8). We could distinguish only a limited number of completely formed Bowman’s glands at E16.5 (data not shown), while at E18.5 and in postnatal mice, numerous Bowman’s glands were present. We did not find TMEM16A expression in Bowman’s glands between E16.5 and P4. However, one study showed expression of TMEM16A in Bowman’s glands in adult mice and rats [16]. As the majority of TMEM16A^-/-^ mice die by P9, our study was limited to P4, and we cannot exclude the possibility that TMEM16A is expressed in Bowman’s glands in adult mice. We found that at P4 nasal septal glands and lateral nasal glands showed a strong TMEM16A immunopositive signal. In both types of glands TMEM16A was expressed in the apical region of secretory acinar cells and in the luminal surface of the glands, where it coexpressed with aquaporin 5 (Fig 8).

In TMEM16A^-/-^ mice, aquaporin 5 immunopositive signals were similar to those observed in TMEM16A^+/+^ mice showing that the morphology of Bowman’s glands, nasal septal glands and lateral nasal glands was not largely modified by the absence of TMEM16A.

## Conclusions

In conclusion, our data provide the first immunohistochemistry study comparing the development of the olfactory epithelium in TMEM16A^+/+^ and TMEM16A^-/-^ littermate mice during embryonic development. We did not find any significant difference in the olfactory epithelium up to P4 between the two types of mice, indicating that TMEM16A does not seem to be involved in proliferation and development of the olfactory epithelium. As TMEM16A^-/-^ mice die soon after birth, preventing functional and behavioral studies in adult mice, the development and use of conditional knockout mice for TMEM16A will allow planning of additional experiments to improve our present knowledge of the function of TMEM16A in the olfactory system.
